# Advances and perspectives in genetically encoded biosensors for biomarker detection and theranostic circuits

**DOI:** 10.1016/j.synbio.2026.09.006

**Published:** 2026-09-23

**Authors:** Jianping Xu, Qingsheng Qi, Qian Wang

**Affiliations:** a National Glycoengineering Research Center, Shandong University, Qingdao, 266237, PR China; b State Key Laboratory of Microbial Technology, Shandong University, Qingdao, 266237, PR China

**Keywords:** Genetically encoded biosensors, Biomarker detection, Synthetic biology, Engineered probiotics, Theranostic circuits

## Abstract

Effective biomarker detection is critical for timely and accurate clinical disease diagnosis. However, conventional analytical methodologies are often constrained by laborious procedures, reliance on specialized equipment, and an inherent inability to perform continuous in situ monitoring. To circumvent these limitations, synthetic biology has pioneered the development of genetically encoded biosensors as a rapid, highly sensitive, and cost-effective alternative. This review specifically focuses on transcriptional regulation-based biosensors, primarily those utilizing allosteric transcription factors (aTFs) and two-component systems (TCSs). By integrating these regulatory sensing modules into engineered microbial chassis, researchers can achieve continuous, real-time biomarker monitoring even within complex and otherwise inaccessible physiological environments. This review particularly focuses on microbial chassis rather than mammalian systems due to their highly tractable genetics, rapid response kinetics, and their unique potential to be deployed as autonomous living probiotics for in situ theranostics. Furthermore, coupling these sensors with engineered actuator genes enables the construction of intelligent therapeutic genetic circuits. This closed-loop theranostic paradigm facilitates simultaneous, real-time diagnosis and autonomous, localized therapeutic interventions, paving the way for personalized and dose-adaptive precision medicine. Finally, we summarize recent advancements in these biosensor platforms, explore their applications in targeted disease interventions, and discuss the contemporary challenges alongside future perspectives for their clinical translation.

## 1 Introduction

Prompt and accurate disease diagnosis represents a cornerstone in optimizing treatment outcomes, typically achieved via the detection of pathognomonic biomarkers [1] (Fig. 1A). Ideal diagnostic modalities demand rapid turnaround, high sensitivity, and robust fidelity. Conventional analytical techniques include polymerase chain reaction (PCR) [2], lateral-flow immunometric assays [3], electrochemical methods [4], DNA sequencing, microfluidics [5,6] and enzyme-linked immunosorbent assays (ELISA) [7]. Despite their utility, these classical frameworks routinely require high-precision instrumentation, cost-prohibitive reagents, intricate sample preparation steps, and time-consuming quantification protocols [8]. Moreover, they remain fundamentally limited in terms of real-time monitoring capabilities and *in vivo* sensitivity. Genetically encoded biosensors offer a compelling alternative by capturing target biomolecular inputs and transducing them into quantifiable output signals, thereby emerging as a promising strategy for biomarker detection. The distinct advantages of these biosensors lie in their capacity for rapid, sensitive, non-invasive, cost-effective, and real-time monitoring [9,10].Typically, a genetically encoded biosensor comprises three interconnected functional modules: a sensing module that detects specific inputs (e.g., metabolites, chemical cues, or environmental stimuli), a regulatory module tasked with signal transduction, and an output module that generates a reporter signal [11].

**Fig. 1:**
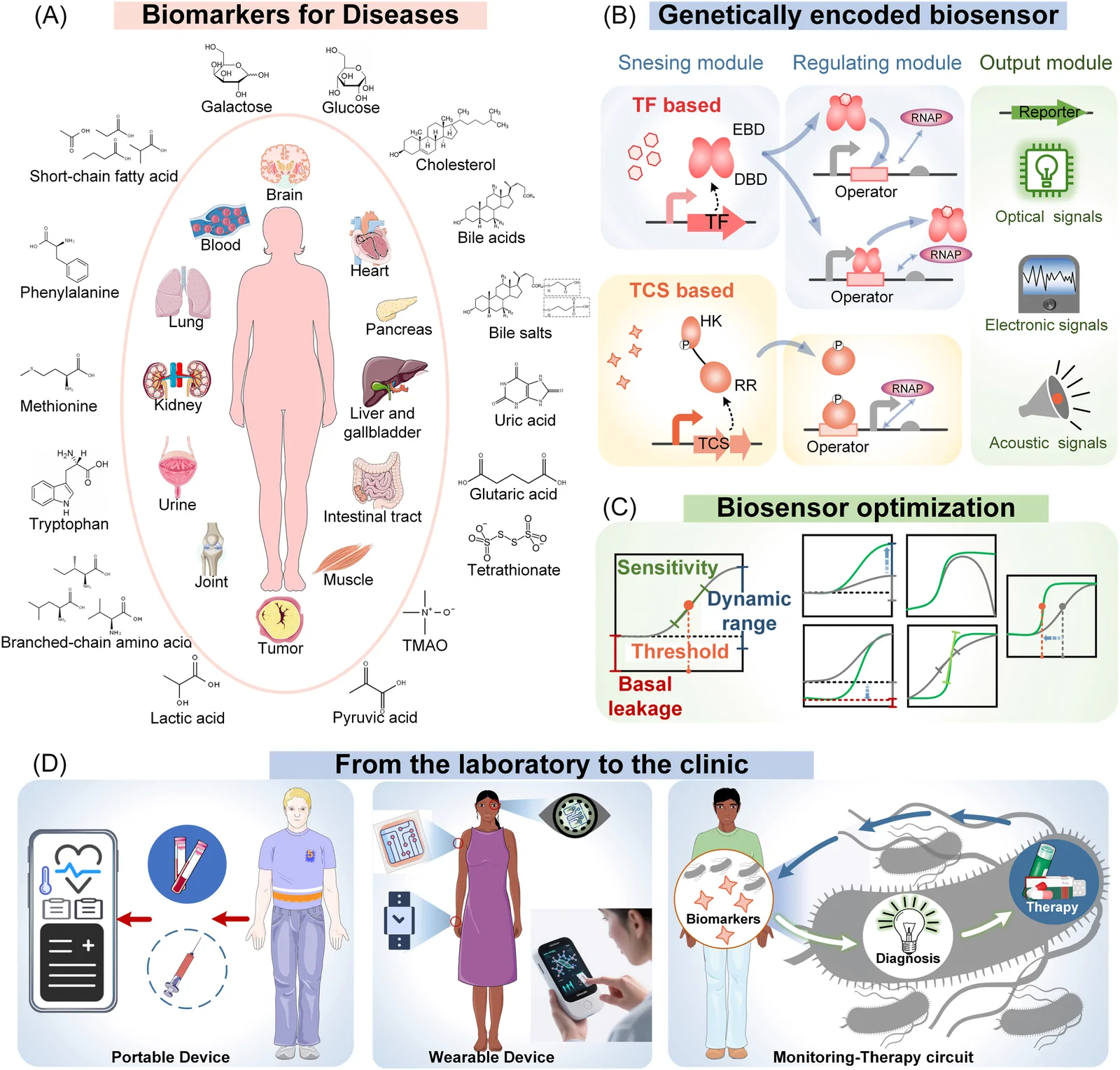
Overview of genetically encoded biosensors for disease biomarker detection and clinical application. (A) Representative diseases and their corresponding biomarkers across various human systems. (B) Core architecture of a genetically encoded biosensor, consisting of sensing, regulating, and output modules. (C) Optimization of biosensor performance parameters such as sensitivity, dynamic range, threshold, and basal leakage, with the goal of tailoring the sensor response curve to meet precise clinical needs. (D) Translational applications from laboratory prototypes to clinical utility, including portable diagnostics, wearable devices, and living microbial platforms."

Genetically encoded biosensors primarily operate via mechanisms such as allosteric transcription factors (aTFs), two-component systems (TCSs), Förster resonance energy transfer (FRET), and nucleic acid-based architectures. FRET- and nucleic acid-based systems have been widely deployed in diagnostics due to their high sensitivity, operational efficiency, and real-time, second-scale monitoring capabilities without latency, which has been comprehensively reviewed elsewhere [[12], [13], [14], [15], [16]]. Nevertheless, transcriptional regulation-based frameworks (specifically aTFs and TCSs) hold unparalleled promise for next-generation intelligent theranostics. Crucially, transcriptional regulation-based biosensors not only possess exceptional signal amplification and high modularity, but also directly couple biomarker detection to sustained functional outputs. This enables their seamless integration into closed-loop “monitoring-treatment” circuits, where biomarker detection drives the autonomous activation of therapeutic modules for disease intervention. While transcriptional circuits inherently encounter limitations including kinetic latency, basal leakage, and host metabolic burden, advanced engineering strategies are steadily mitigating these drawbacks, reinforcing their role as a cornerstone of intelligent genetic circuits. Due to this distinctive advantage of aTF- and TCS-based biosensors, this review focuses on the current development and emerging potential of these two classes of biosensors for the detection of disease relevant biomarkers. Characteristically, an aTF modulates target gene expression by governing the binding of RNA polymerase to DNA, structurally mediated by a DNA-binding domain (DBD) and an effector-binding domain (BD). Conversely, a classical TCS consists of a membrane-bound histidine kinase (HK), featuring an extracytoplasmic N-terminal stimulus-sensing domain and a cytosolic C-terminal autokinase domain, and a cytosolic response regulator (RR). Upon activation, the HK catalyzes its own autophosphorylation and sequentially transfers the phosphoryl group to the RR, which subsequently regulates gene expression (Fig. 1B). The performance of these genetically encoded biosensors can be quantitatively benchmarked via dose-response profiles (Fig. 1C). To satisfy diverse clinical requirements, key biophysical parameters—including reporter expression intensity, substrate binding affinity, and DNA binding affinity—can be rationally tuned to tailor operational sensitivity and specificity [17].

When deployed within an appropriate host organism, genetically encoded biosensors unlock powerful capabilities, notably continuous *in vivo* surveillance. While mammalian cell-based systems have made significant progress in biosensing, this review primarily focuses on microbial chassis (such as engineered bacteria and yeast). Microbial platforms are predominantly utilized for aTF- and TCS-based circuits due to their extensive genetic toolkits for complex circuit wiring, robust environmental adaptability, and cost-effectiveness. More importantly, unlike mammalian cells, by colonizing engineered microbes within the gastrointestinal tract or systemic circulation, target biomarkers can be monitored continuously without the need for invasive, longitudinal sample collection. For instance, Mimee et al. [18] developed an engineered *Escherichia coli* Nissle 1917 (EcN) strain integrated into an ingestible micro-bio-electronic device to detect intestinal bleeding. Upon localized hemorrhage, the engineered EcN absorbs luminal heme, which is sensed by the intramicrobial HrtR biosensor, ultimately triggering a quantifiable optical signal for real-time monitoring. Similarly, Danino et al. [19] established an oral diagnostic strategy using engineered EcN that translocates to hepatic and colorectal tumors; these microbes detect metastatic niches and generate a luminescent output shed into the urine, enabling non-invasive detection within 24 h. Furthermore, owing to their micrometer-scale dimensions, these living biosensors can readily access anatomically sequestered environments, such as the deep lumen of the small intestine or the necrotic center of solid tumors, dramatically accelerating biomarker accessibility [20,21]. The spatial proximity between engineered biosensors and the site of biomarker generation also empowers these living platforms to exert localized therapeutic effects. These diagnostic circuits can be expanded by incorporating genetic modules that facilitate autonomous drug synthesis, controlled secretion, or the metabolic clearance of pathological toxins. Indeed, EcN is already widely used as a probiotic chassis to treat diseases. Leventhal et al. [20] leveraged EcN as a living cell platform for targeted cancer therapy, while Zhao et al. [22] engineered a highly efficient uric acid degradation pathway in it to successfully mitigate hyperuricemia and gout risk in murine models. In recent years, such intelligent gene circuits have also advanced into clinical trials for metabolic disorders. For example, phenylketonuria (PKU) is an inherited metabolic disease where toxic phenylalanine accumulation induces severe neurotoxicity. To address this, Isabella et al. [21] engineered EcN to sense the hypoxic colonic environment and actively drive the expression of phenylalanine-metabolizing genes, converting excess systemic phenylalanine into non-toxic metabolites (cinnamic acid and phenylpyruvic acid) for urinary excretion. In another therapeutic context, intestinal inflammation generates reactive oxygen species that react with endogenous thiosulfate in the gut, resulting in the formation of tetrathionate [23]. By coupling a tetrathionate-responsive biosensor to a microcin production circuit, engineered bacteria selectively synthesize antimicrobials only at inflamed sites, effectively inhibiting *Salmonella* colonization and ameliorating inflammatory bowel disease [24].

In summary, genetically encoded biosensors offer a transformative strategy for early-stage disease diagnosis and longitudinal physiological monitoring, shifting healthcare paradigms from conventional centralized hospital settings to portable, decentralized, and daily-life diagnostics. Rational integration of regulatory modules with therapeutic actuator genes enables the realization of real-time, closed-loop theranostic circuits. This next-generation paradigm tightly couples autonomous biomarker sensing with localized, responsive drug delivery. By restricting therapeutic intervention strictly to the pathological microenvironment, such systems hold immense potential to minimize systemic adverse effects, support dynamically adjustable dosage regimens, and ultimately advance personalized precision medicine (Fig. 1D). Given the accelerating breakthroughs and expanding clinical horizons of genetically encoded biosensors, this review provides a comprehensive synthesis of recent advances in both established and emerging biosensing platforms. Furthermore, we critically analyze the contemporary challenges and future directions necessary for their sustained development and successful clinical translation.

## 2 Biomarkers for different diseases and their biosensors

Organized according to a biomarker property classification framework, this review offers a comprehensive analysis of disease associated biomarkers and the corresponding genetically encoded biosensors engineered for their detection. A concise summary of these platforms is presented in Table 1.

**Table 1:** Summary of disease relevant biomarkers and the corresponding genetically encoded biosensors engineered for their detection.

Classification	Biomark	Related Disease	Type	Component	Application	Output	Chassis	Validation stage	Existing detection method	Ref.
Carbohydrates	Glucose	Diabetes	TCS	CpxAR	Clinical diagnosis	Fluorescence	*E*. *coli*	Ex vivo proof-of-concept	Electrochemical Biosensor	[25]
			aTF	HexR	Clinical theranostics	Glucagon-like peptide 1	*E*. *coli*	In vivo proof-of-concept		[26]
	Galactose	Galactosemia	aTF	Gal4p	Metabolic engineering	Gene Expression	*S*. *cerevisiae*	-	HPLC, LC-MS	[27]
Amino acids	Phenylalanine	Phenylketonuria	aTF	PAH regulatory domain	Clinical theranostics	Phe degrading enzymes	Mammalian cell	In vivo proof-of-concept	HPLC, LC-MS	[28]
	Branched-chain amino acids	Obesity-related metabolic disorders	aTF	Leu3p	Metabolic engineering	Fluorescence	*S*. *cerevisiae*	-	Enzymatic Method	[29]
	Tryptophan	Neurological disorders (depression, Alzheimer's disease)	aTF	TrpR	Metabolic engineering	Fluorescence	*E*. *coli*	-	ELISA	[[30], [31], [32], [33]]
	Methionine	homocystinuria	aTF	Lrp	Metabolic engineering	Fluorescence	*C*. *glutamicum*	-	FRET, Atomic Absorption Spectroscopy	[34]
Steroids	Cholesterol	Atherosclerosis; cardiovascular diseases; Tumors	aTF	SREBP	-	-	-	-	Enzymatic Method	[35]
	Bile acids	Colorectal cancer; liver cancer, genetic diseases, obesity, diabetes, inflammation	aTF	BreR,VFA0359	Clinical diagnosis	Fluorescence	*B*. *thetaiotaomicron*	Ex vivo proof-of-concept	ELISA	[[36], [37], [38]]
	Bile salts	Early-stage liver cirrhosis, hepatitis, drug-induced liver injury	aTF	TcpP-CadC, VtrA-CadC	Clinical diagnosis	Colorimetric output	*E. coli*	Ex vivo proof-of-concept	Enzymatic Method	[[39], [40], [41]]
Short-chain fatty acids	Formate	Gut microbiome ecosystem-associated diseases	aTF	FhlA	Metabolic engineering	enzyme activity	*E. coli*	-	GC-MS, LCMS	[42]
	Acetate		aTF	Haa1- BM3R1	Metabolic engineering	Fluorescence	*S*. *cerevisiae*	-		[43]
	Propionate		aTF	War1p,PrpR	Metabolic engineering	Fluorescence	*S*. *cerevisiae*	-		[44]
	Butyrate		TCS	BumSR	-	-	-	-		[45]
	Isobutyrate		aTF	War1p	Metabolic engineering	Fluorescence	*S*. *cerevisiae*	-		[46,47]
	Isovalerate									
	Caproate									
Other metabolism disorder-related compounds	Pyruvic acid	Cancer; tumors	aTF	PdhR	-	Gene Expression	*E. coli*	-	Enzymatic Method	[48,49]
	Lactic acid		aTF	LldR	Metabolic engineering	Luminescence	*E*. *coli*	-		[50,51]
	Glutaric acid	Glutaric Aciduria	aTF	CsiR	Clinical diagnosis	Fluorescence	Mammalian cell	Ex vivo proof-of-concept	GC-MS, LCMS	[52]
	Uric acid	Gout, hyperuricemia; kidney diseases	aTF	HucR	Clinical theranostics	Uric acid degrading enzymes	Mammalian cell	In vivo proof-of-concept	Enzymatic Method, Dry Chemistry Method	[53,54]
	Trimethylamine N-oxide	Cardiovascular disease risk, chronic kidney disease risk;	TCS	TorRST	Clinical theranostics	TMAO degrading enzymes	*E. coli*	Ex vivo proof-of-concept	ELISA	[55]
	Tetrathionate	Intestinal inflammation	TCS	TtrSR	Clinical theranostics	Tetrathionate reductase	*E. coli*	Ex vivo proof-of-concept	Iodometric Titration, Ion Chromatography	[56,57]
Quorum sensing signals	AHLs	Cystic Fibrosis; inflammatory bowel disease	aTF	TraR	Clinical diagnosis	Luminescence	*A*.*tumefaciens*	Ex vivo proof-of-concept	LC-MS	[58]

General chromatographic and mass spectrometric techniques such as High-Performance Liquid Chromatography, Liquid Chromatography-Tandem Mass Spectrometry, and Gas Chromatography-Mass Spectrometry are abbreviated or generalized as HPLC/LC-MS/GC-MS, and only specific or unique conventional methods are highlighted for individual biomarkers.

### 2.1 Carbohydrate

#### 2.1.1 Glucose

Diabetes mellitus is a prevalent metabolic disorder characterized by chronic hyperglycemia, which drives severe cardiovascular, renal, and ocular complications. Consequently, continuous and dynamic glucose surveillance is paramount for personalized precision interventions. While the current diagnostic landscape is dominated by electrochemical sensors reliant on glucose oxidase [59], which, although sensitive, suffer from limited stability, a short lifespan [59,60], and an inherent inability to integrate with autonomous therapeutic actuators.

Genetically encoded biosensors offer a transformative alternative by exploiting cellular regulatory networks. In *Escherichia coli*, the CpxAR system can be repurposed for glucose sensing: ligand activation triggers the sensor kinase CpxA to phosphorylate the response regulator CpxR, which drives downstream transcription from promoters like P_*cpxP*_. This architecture enables the conversion of extracellular metabolic signals into programmable genetic outputs [61]. Alexis et al. [25] engineered a CpxAR responsive biosensor with a detection limit of 0.1 mM, outperforming conventional urine strips. By incorporating genetic logic gates and signal amplification circuits, this platform achieved robust signal processing and memory storage. When encapsulated in hydrogel beads, the engineered microbes successfully detected pathological glucose levels in human urine (Fig. 2A), establishing a vital precedent for autonomous diagnostic and therapeutic systems. Alternatively, the transcriptional regulator HexR from *Pseudomonas putida* offers another strategy for glucose sensing. Guan et al. [26] engineered *Escherichia coli* Nissle 1917 to develop a glucose-sensing and functional response probiotic living drug (GIFT), which triggers glucagon-like peptide 1 (GLP-1) expression in response to high glucose levels and terminates production upon normalization. This closed-loop system demonstrated robust therapeutic efficacy in controlling blood glucose across multiple diabetic mouse and non-human primate models.

**Fig. 2:**
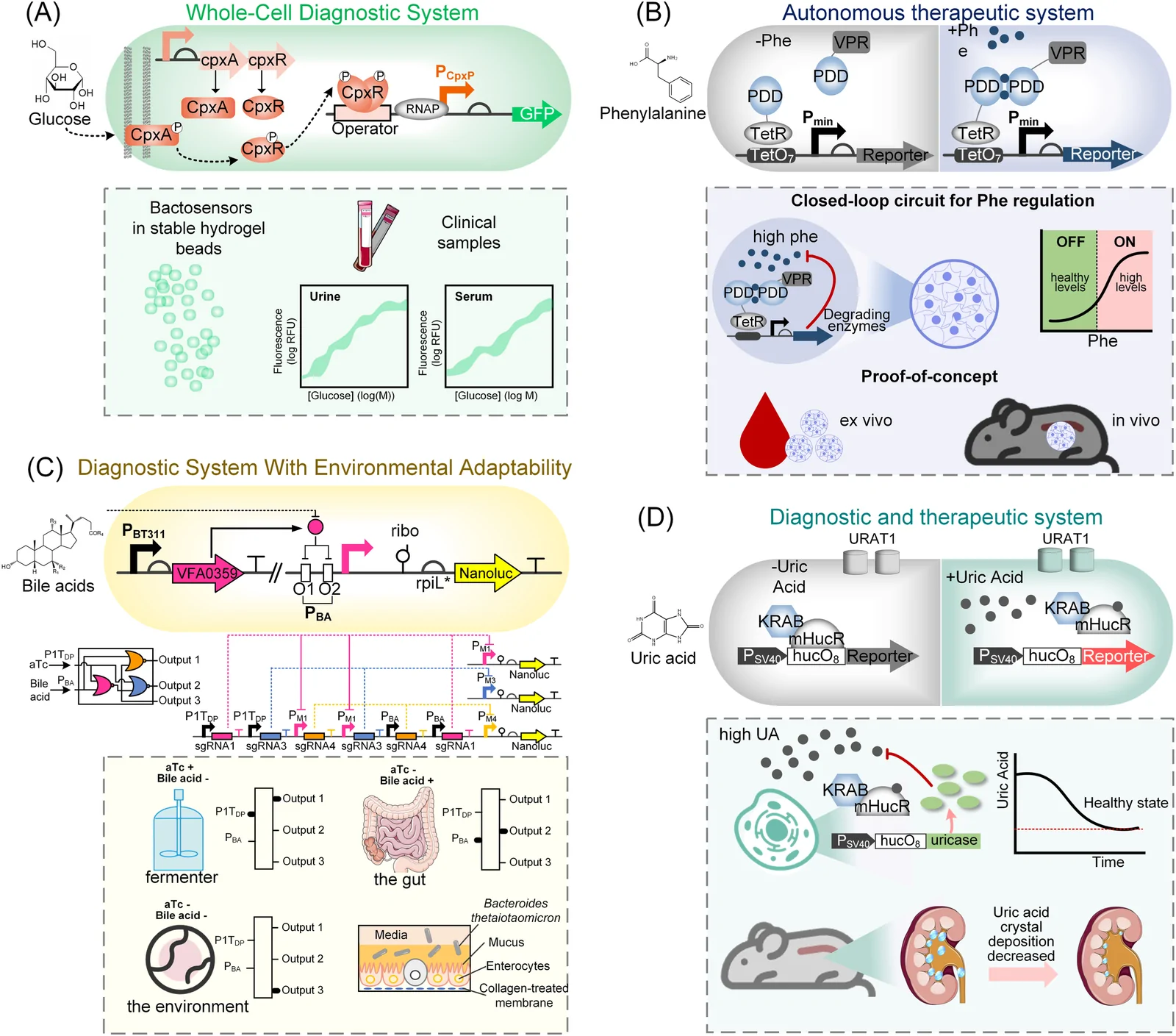
Engineered biosensors for biomarker diagnosis and theranostic circuits. (A) CpxAR-based glucose diagnostic system. Glucose triggers CpxA kinase activity and subsequent CpxR phosphorylation ("P"), driving target gene transcription. Solid arrows denote protein expression; dashed arrows indicate CpxAR signal transduction and allostery. Hydrogel encapsulation enables glucose quantification in clinical samples. (B) Phenylalanine-responsive theranostic circuit. Phenylalanine-induced dimerization of a human PAH-derived module activates phenylalanine-degrading enzymes. Alginate-encapsulated cells effectively reduce toxic metabolites in human blood and mouse models. (C) A diagnostic system for bile acids with environmental adaptability. Utilizing the VFA0359 regulator and genetic logic gates, engineered bacteria specifically activate in the mammalian gut while remaining dormant in external environments. (D) A theranostic circuit utilizing the HucR repressor. Uric acid binding induces dissociation of the KRAB-HucR fusion protein, triggering signal output and uricase production, which maintains healthy serum uric acid levels and reduces renal crystal deposition in mouse models.

#### 2.1.2 Galactose

Galactose is the pathognomonic biomarker for galactosemia, an inherited metabolic disorder where congenital enzymatic deficiencies induce systemic galactose toxicity. While early newborn screening is vital, conventional chromatographic and enzyme assays remain restricted by their reliance on specialized equipment, failing to support continuous or decentralized point of care monitoring.

The transcription factor Gal4p from *Saccharomyces cerevisiae* provides a well-established platform for galactose sensing [27]. Upon binding galactose, Gal4p activates downstream promoters such as *GAL1*, *GAL7*, and *GAL10*, enabling strong and tunable transcriptional responses. This system has been widely applied in synthetic biology as an inducible gene expression module and serves as a robust foundation for constructing genetically encoded galactose biosensors [62]. Although Gal4p mediated systems yield a high dynamic range and robust tunability, their application remains largely restricted to engineered laboratory models. To date, the galactose biosensor has not been deployed for clinical diagnostics. Key translational challenges include adapting these sensors to physiological environments, mitigating background leakage, and integrating them into clinically relevant platforms for real time monitoring.

### 2.2 Amino acid

#### 2.2.1 Phenylalanine

Phenylalanine (Phe) is a key biomarker for phenylketonuria (PKU), a genetic metabolic disorder caused by phenylalanine hydroxylase deficiency, leading to toxic accumulation of Phe in blood and brain [63]. Elevated Phe levels can result in neurological dysfunction, emotional and cognitive impairments, and severe intellectual disability, particularly in children. Therefore, tight regulation of blood Phe levels is essential for effective disease management [64,65]. Current diagnosis and monitoring rely on tandem mass spectrometry [66], which, although accurate, lacks the capacity for dynamic or real time surveillance.

To address this, Isabella et al. engineered the probiotic strain SYNB1618 by integrating phenylalanine ammonia lyase into EcN to metabolize excess phenylalanine directly within the gastrointestinal tract, successfully clearing dietary and circulating intestinal Phe in preclinical models. Subsequently, an optimized strain, SYNB1934, was developed via directed evolution of the phenylalanine ammonia-lyase enzyme, yielding enhanced phenylalanine degradation activity. Notably, both SYNB1618 and SYNB1934 have demonstrated highly positive outcomes in Phase 2 clinical trial [67,68]. However, this open loop architecture lacks adaptive feedback control. To achieve autonomous regulation, Galvan et al. developed the Phenylalanine Responsive Off switch (PRO), a biosensor circuit where elevated Phe drives human PAH domain dimerization to actively trigger the expression of Phe degrading enzymes [28,69]. Encapsulated in alginate microcapsules, PRO cells effectively reduced Phe concentrations ex vivo in human blood and *in vivo* in murine models (Fig. 2B).

#### 2.2.2 Branched-chain amino acids

Elevated circulating branched chain amino acids (BCAAs)—leucine (Leu), isoleucine (Ile), and valine (Val)—are strongly associated with insulin resistance, obesity [70], and confer up to a fivefold increased risk of type 2 diabetes [71,72]**,** despite their physiological role in activating mTORC1 to promote protein synthesis. Current detection methods are largely based on mass spectrometry and are not suitable for real-time or continuous monitoring, limiting their utility in dynamic metabolic assessment and personalized intervention [72,73].

Genetically encoded biosensors constitute a powerful tool for the dynamic assessment of BCAA fluxes. In yeast, the transcription factor Leu3p regulates BCAA biosynthesis by sensing alpha keto acid intermediates, including α-isopropylmalate (α-IPM), α-ketoisovalerate (α-KIV), and α-keto-3-methylvalerate (α-K3MV), that directly correlate with Leu, Val, and Ile levels, shifting from a repressor to an activator upon metabolite binding [[74], [75], [76]]. Utilizing this mechanism, Zhang et al. constructed a Leu3p-based biosensor to report intracellular BCAA metabolic fluxes [29]. The Leu3p-based system highlights the potential of leveraging endogenous metabolic regulators to indirectly sense clinically relevant metabolites through pathway intermediates. Such strategies may enable real-time monitoring of metabolic states, treatment responses, and microbiome contributions to host metabolism. However, the clinical translation requires addressing cross reactivity among branched chain intermediates, refining operational specificity, and validating performance within complex physiological fluids.

#### 2.2.3 Tryptophan

Tryptophan (TRP) metabolism via the kynurenine (KYN) pathway regulates serotonin and melatonin production [77]. Dysregulation of TRP metabolism drives neuroinflammation, immune evasion, and malignancy [[78], [79], [80], [81], [82]]. Consequently, the KYN/TRP ratio serves as a vital biomarker for disease progression [78,83,84]. However, current detection methods rely mainly on chromatography-based techniques or immunoassays, which require specialized equipment and are not suitable for real-time or in situ monitoring [85]. The *Escherichia coli* tryptophan repressor (TrpR) provides a direct sensing platform: upon binding tryptophan, TrpR homodimerizes to target the *trpO* operator, actively repressing downstream aromatic metabolism genes [30,31,85]. This ligand-dependent regulatory mechanism provides a direct and highly specific sensing module for intracellular TRP levels.

Exploiting this direct factor mediated mechanism, Gong et al. engineered a series of TrpR variants with tunable dynamic ranges and improved ligand selectivity to expand its applicability [32], while Li et al. employed it for screening efficient tryptophan-metabolizing enzymes, demonstrating its robustness in directed evolution workflows [33]. The consistent performance of TrpR across these applications highlights its potential as a versatile sensing module for *in vivo* tryptophan detection. Despite their success in pathway engineering, clinical translation of TrpR platforms demands mitigating host microenvironment interference, expanding the narrow *in vivo* dynamic range, and coupling these modules with real time diagnostic outputs.

#### 2.2.4 Methionine

Methionine is an essential amino acid involved in protein synthesis and interconnected carbon, nitrogen, and sulfur metabolism, and serves as a precursor for biologically active molecules such as polyamines [86,87]. Clinically, elevated blood methionine is a key biomarker for homocystinuria, a metabolic disorder caused by cystathionine β-synthase deficiency, where methionine levels can increase by over 100 μM [88]. Accurate quantification of methionine is therefore critical for diagnosis and therapeutic monitoring. Current detection methods, including chromatography [89], atomic absorption spectroscopy [90], capillary electrophoresis–mass spectrometry [91], enzymatic assays [92], and fluorescence-based techniques [93], provide high sensitivity but are often time-consuming, labor-intensive, and unsuitable for real-time or point-of-care applications.

Genetically encoded biosensors offer a promising alternative for dynamic methionine detection. In *Corynebacterium glutamicum*, the transcriptional regulator Lrp controls the expression of the *brnFE* operon, which encodes an amino acid efflux pump responsible for exporting excess intracellular amino acids [94,95]. Mustafi et al. developed an Lrp-based whole-cell biosensor where methionine activated Lrp induces an eYFP fluorescent reporter, a design validated in high throughput strain screening [34]. The Lrp-based system demonstrates the feasibility of leveraging native amino acid-responsive regulators for constructing methionine biosensors with relatively simple architectures and robust outputs. Although this Lrp mediated strategy provides robust, simplified architectures, transitioning from industrial settings to *in vivo* clinical monitoring necessitates widening the dynamic range, optimizing specificity, and validating performance within complex physiological environments.

### 2.3 Steroid compounds and their derivatives

#### 2.3.1 Cholesterol

Dysregulated cholesterol metabolism drives a wide spectrum of systemic pathologies, encompassing cardiovascular, oncological, and neurological disorders [96]. Critically, elevated low density lipoprotein cholesterol (LDL-C) serves as a primary clinically established biomarker for atherosclerosis and heart disease [97]. Beyond vascular contexts, aberrant cholesterol dynamics accelerate tumor progression, positioning this sterol as both a diagnostic indicator and therapeutic target [98,99]. Although conventional enzymatic, electrochemical, and chromatographic assays offer high sensitivity, their translation to real time or point of care monitoring remains bottlenecked by logistical complexities, expensive instrumentation, and specialized labor requirements, limiting their applicability for real-time and point-of-care monitoring [100,101].

To bridge this gap, natural homeostatic frameworks can be leveraged for biosensor development. Sterol regulatory element binding proteins (SREBPs) ordinarily govern cholesterol biosynthesis and uptake genes (e.g., *HMGCR* and *LDLR*) by targeting promoter sterol regulatory elements (SREs) [102]. Intracellular cholesterol directly modulates this cascade: elevated sterol levels prevent the maturation of endoplasmic reticulum bound SREBP precursors, suppressing downstream transcription [35]. This endogenous feedback mechanism enables coupling between cholesterol levels and gene expression outputs. Leveraging this property, SREBP–SRE regulatory modules can be repurposed as cholesterol-responsive biosensors to dynamically report intracellular cholesterol status. SREBP-based systems offer a physiologically relevant sensing strategy that inherently integrates cholesterol detection with endogenous regulatory networks, providing opportunities for coupling biosensing with therapeutic interventions. In principle, such circuits can couple with actuators that inhibit *HMGCR*, modulate *PCSK9*, enhance *LDLR*, or regulate intestinal Cholesin to achieve autonomous closed loop control of cholesterol homeostasis [103]. However, clinical translation demands overcoming liabilities related to pathway cross talk, complex feedback logic, and *in vivo* operational robustness.

#### 2.3.2 Bile acid

Bile acids are cholesterol derived sterol metabolites essential for lipid digestion, bile secretion, and metabolic homeostasis [104]. Aberrant bile acid profiles strongly correlate with colorectal and hepatic malignancies, metabolic syndrome, and localized inflammation [[105], [106], [107], [108]]. Particularly, deoxycholic acid (DCA) represents a major colonic fraction and a vital biomarker for hepatobiliary and intestinal diseases [[109], [110], [111], [112]]. Despite this high diagnostic and prognostic value, current analytical pipelines lack the portability required for real time, portable, or in situ monitoring.

Advanced genetically encoded platforms have successfully translated native bacterial regulators into responsive theranostic frameworks. Beabout et al. utilized the transcription factor BreR from *Bacteroides fragilis* within paper-based cell-free expression systems to selectively detect bile acids across complex fecal water, wastewater, and serum matrices [36]. Similarly, Taketani et al. harnessed the regulator VFA0359 from *Vibrio cholerae* to engineer a real time sensing circuit in *Bacteroides thetaiotaomicron*, enabling real-time sensing of bile acids [37]. Importantly, this sensing module was integrated into a genetic circuit, conferring a “sense-and-respond” capability in gut-mimicking environments. Engineered bacteria adhered to intestinal epithelial cells and released therapeutic molecules specifically under elevated bile acid conditions, while maintaining biosafety through controlled expression, efficient growth in production settings, and self-inactivation upon excretion (Fig. 2C). Furthermore, directed evolution of these responsive repressors has expanded sensor specificity: engineered BreR variants achieved high selectivity for DCA and successfully unlocked the detection of ursodeoxycholic acid (UDCA), a clinically vital therapeutic bile acid unrecognized by the wild type counterpart [38]. Bile acid-responsive systems illustrate the integration of biosensing with environmental adaptability and therapeutic output. The ability to engineer specificity toward individual bile acid species further expands their diagnostic and therapeutic utility. Despite these breakthroughs, managing bile acid structural diversity, minimizing cross reactivity, and ensuring precise *in vivo* control remain key hurdles.

#### 2.3.3 Bile salts

Bile salts, formed via glycine or taurine conjugation, undergo active biliary transport through ABCB11 [113]. Clinically, bile salts serve as sensitive biomarkers for liver function and are widely used in the diagnosis of liver diseases, including cirrhosis, hepatitis, drug-induced liver injury, and intrahepatic cholestasis of pregnancy [114,115]. Their levels dynamically reflect biliary obstruction and therapeutic response, making them valuable for disease monitoring [116]. However, current diagnostic approaches rely on multi-marker panels and centralized laboratory testing, which are often limited by low specificity at early stages, delayed response, and lack of accessibility for continuous monitoring [117,118]. Given the chronic and progressive nature of hepatobiliary diseases, rapid and continuous monitoring tools are urgently required [39].

Pathogenic survival mechanisms have inspired high fidelity diagnostic platforms. In *Vibrio* species, ambient bile salts act as environmental cues that trigger virulence cascades via membrane bound sensory complexes, including TcpP-TcpH and VtrA-VtrC [40,41]. Exploiting these architectures, Chang et al. [39] established the modular EMeRALD platform, which fuses a universal signaling scaffold with interchangeable ligand binding domains (LBDs). Bile salt binding induces receptor dimerization, driving downstream gene expression. By incorporating TcpP or VtrA modules alongside their native stabilizing factors TcpH or VtrC, the system achieved robust signal transduction [119]. Following directed evolution and output optimization, EMeRALD successfully quantified bile salts in patient serum, accurately identifying abnormal concentrations exceeding 37 μM with visible outputs that matched standard enzymatic assays. This platform demonstrates the potential of modular receptor engineering to construct customizable biosensors with clinical relevance. Its ability to repurpose pathogen-derived sensing mechanisms into safe diagnostic tools highlights a promising strategy for point-of-care monitoring. Nevertheless, further optimization is required to enhance specificity, reduce background noise, and ensure robustness across diverse physiological conditions.

#### 2.3.4 Short-chain fatty acids

Short-chain fatty acids (SCFAs)—primarily acetate, propionate, butyrate, and formate—are microbial fermentation products crucial for host metabolic and immune health [[120], [121], [122]]. Alterations in SCFA levels are closely associated with metabolic and immune-related diseases, and fecal SCFAs are widely recognized as biomarkers reflecting gut microbiota composition and host metabolic status [123]. Since standard analytical techniques lack continuous in situ surveillance capabilities [124,125], diverse transcription factors and two-component systems have been harnessed for real-time SCFA detection.

For formate, In *Escherichia coli*, formate metabolism is regulated by the σ^54^-dependent transcriptional activator FhlA, which controls the *fdnGHI* operon. Wang et al. constructed a biosensor based on FhlA and its cognate promoter P_*fdhF*_, achieving a linear detection range from 1.0 to 250 mM [42]. For acetate, Mormino et al. engineered a synthetic biosensor by combining the *Saccharomyces cerevisiae* transcription factor Haa1 [[126], [127], [128], [129]], which responds to weak acid stress and translocates to the nucleus upon acetate binding, with the heterologous regulator BM3R1 to control reporter expression [43]. The resulting system exhibited a linear response in the range of 10–60 mM. Additionally, the transcriptional regulators AlsR from *Bacillus subtilis* and PrpR from *Corynebacterium glutamicum* offer viable alternative modules [130]. For propionate, In yeast, the transcription factor War1p responds to propionate and activates expression of the efflux pump gene *PDR12* [131]. Based on this mechanism, Williams et al. developed a propionate biosensor using War1p and the P_*PDR12*_ promoter, achieving a strong correlation between propionate concentration and reporter output [44]. System optimization using CRISPR-Cas9 increased signal output by ∼10-fold over a concentration range of 0.01–1 mM.

Conversely, a dedicated butyrate biosensor has not yet been fully developed, though the BumSR two-component system from *Campylobacter jejuni* presents a promising engineering foundation [45]. For broader organic acid profiling, War1p-mediated platforms have been expanded to detect medium- and branched-chain fatty acids. Baumann et al. engineered a sensor for short and medium chain fatty acids, including caproate [46], while Miyake et al. achieved selective detection of short branched chain fatty acids (e.g., isovalerate and isobutyrate) by pairing the War1p P_*PDR12*_ node with Pdr12p transporter engineering [47]. Fine tuning *PDR12* expression yielded a 30 to 50-fold fluorescence increase.

In summary, SCFA biosensors highlight the versatility of leveraging both single metabolite and broad-spectrum regulatory networks. While promoter engineering, synthetic feedback loops, and transporter manipulation allow precise tuning of sensitivity and dynamic range, critical translational bottlenecks remain. Specifically, while biosensors for formate, acetate, and propionate possess mature sensing modules with quantifiable dynamic ranges, dedicated detection systems for critical metabolites like butyrate remain underdeveloped. Therefore, the *de novo* design of butyrate-specific biosensors and the achievement of strict specificity among structurally analogous short-chain fatty acids represent major directions for future research and clinical translation.

### 2.4 Other compounds related to metabolic disorders

#### 2.4.1 Pyruvate and lactate

The Warburg effect defines the metabolic reprogramming of cancer cells toward enhanced glycolysis, driving the aberrant accumulation of pyruvate and lactate even under normoxic conditions [132]. Consequently, both metabolites serve as crucial biomarkers for neoplastic diagnosis, staging, and metabolic profiling [133]. Pyruvate, a pivotal intermediate intersecting carbohydrate, lipid, and amino acid pathways [134], is typically maintained at 40 to 120 μM in healthy individuals [135,136] but escalates sharply across various malignancies, showing up to a twofold elevation in gastric cancer [137]. Notably, pyruvate-based detection shows strong diagnostic performance (sensitivity 90.9%, specificity 70%), outperforming traditional serum markers like such as CA19-9 and CEA [[138], [139], [140]]. Concurrently, lactate, produced from pyruvate via aerobic glycolysis, serves as a key indicator of tumor hypoxia and glycolytic activity [[141], [142], [143], [144]]. While dynamic fluctuations in lactate track longitudinal treatment responses—such as dose dependent declines post radiotherapy [145,146]—standard quantification techniques like chromatography, spectrophotometry, and nuclear magnetic resonance remain constrained by high costs, operational complexity, and an inability to provide real time tracking [[147], [148], [149], [150]].

**Pyruvate sensing:** In *Escherichia coli*, the transcriptional regulator PdhR functions as a central controller of pyruvate metabolism by directly sensing pyruvate and regulating the expression of the pyruvate dehydrogenase complex (PDHc) and respiratory chain genes such as *ndh* and *cyoABCDE* [151]. Beyond this, PdhR acts as a global regulator, activating fatty acid β-oxidation genes (*fadE*, *fadI*, *fadJ*) while repressing motility-related genes (*fliAZ*), thereby coordinating metabolic and physiological states [48]. Zou et al. successfully developed a pyruvate-responsive biosensor system based on PdhR for dynamically regulating metabolic flux distribution in central metabolism [48]. Furthermore, Yang et al. extended PdhR functionality into eukaryotic systems, constructing both activation and repression circuits responsive to pyruvate, enabling bidirectional control of gene expression [49]. These studies highlight PdhR as a versatile sensing module with potential applications ranging from metabolic engineering to diagnostic and therapeutic systems.

**Lactate sensing:** While fluorescent protein- and FRET-based lactate sensors have been widely used for biochemical assays and live-cell imaging [152,153], transcription factor-based systems offer greater flexibility for constructing genetic circuits. The allosteric transcription factor LldR from *Pseudomonas aeruginosa* regulates the *lldPDE* operon involved in lactate utilization. In the absence of lactate, LldR binds to the promoter and represses transcription; upon lactate binding, it undergoes conformational changes and dissociates from DNA, thereby activating downstream gene expression [50]. Based on this mechanism, Xiao et al. developed an LldR-based lactate biosensor capable of quantitatively monitoring lactate production in engineered *Klebsiella oxytoca* [51]. The robust responsiveness and regulatory flexibility of LldR make it a promising module for applications in clinical detection as well as in the construction of “sense-and-respond” therapeutic circuits targeting tumor metabolism.

Collectively, pyruvate and lactate biosensors capture distinct, complementary layers of oncological metabolism, reflecting central flux dynamics and tumor microenvironment status, respectively. Transcription factor-based systems such as PdhR and LldR further enable integration of sensing with gene regulation, offering opportunities for closed-loop therapeutic applications. While these allosteric modules enable autonomous, closed loop therapeutic architectures, clinical translation requires mitigating background matrix interference within complex samples, optimizing detection limits across physiological ranges, and validating long term *in vivo* stability.

#### 2.4.2 Glutaric acid

Glutaric acid (GA) is a dicarboxylic intermediate in lysine and tryptophan catabolism, normally degraded by mitochondrial glutaryl CoA dehydrogenase (GCDH). Congenital GCDH deficiency causes glutaric aciduria type I (GA1), an inherited metabolic disease marked by toxic GA accumulation in tissues and biological fluids, precipitating acute, stress induced neurotoxic crises [[154], [155], [156]]. Current diagnostic gold standards, such as GC/MS and MS/MS, rely on static, ex vivo sampling and cannot capture real time metabolic fluctuations or provide early warnings of impending crises, emphasizing the need for responsive, in situ biosensors.

To address this, the GntR family transcriptional repressor CsiR from *Pseudomonas putida* KT2440—which natively regulates the glutamate hydroxylation operon csiD lhgO—has been repurposed for GA sensing [[157], [158], [159]]. Gao et al. developed a high performance genetically encoded GA biosensor, termed Glusor, by executing systematic domain truncations, insertion site screenings, and linker engineering on CsiR [52]. Glusor delivers high specificity, rapid reversibility, and robust stability, establishing a low cost, high throughput screening alternative to mass spectrometry for newborn screening and clinical GA1 management. Beyond diagnostics, subcellularly targeted Glusor variants allow the non-invasive visualization of compartmentalized GA dynamics and metabolic heterogeneity without radioisotope labeling. Despite its strong performance, further work is required to validate Glusor in complex physiological and clinical settings, improve *in vivo* quantitative accuracy, and integrate it into closed-loop systems for early warning and intervention in metabolic crises. Nevertheless, this approach represents a shift from static, ex vivo diagnostics toward dynamic, real-time metabolic monitoring.

#### 2.4.3 Uric acid

Uric acid is the terminal product of purine catabolism and a primary plasma antioxidant that scavenges reactive oxygen species to mitigate oxidative damage [[160], [161], [162], [163]]. However, systemic hyperuricemia causes urate supersaturation, driving gouty inflammation [164,165], renal dysfunction [166,167], and broader oxidative stress related pathologies in cardiovascular disease and oncology [53,168]. Given its central role in disease, uric acid serves as an important clinical biomarker for diagnosis and monitoring. Standard clinical assays rely on intermittent, destructive tracking and lack the capacity for real time feedback regulation or continuous disease management.

To overcome this, researchers have harnessed HucR, a ligand responsive repressor from *Deinococcus radiodurans* that dissociates from its operator to activate a putative uricase operon upon uric acid binding [54]. Kemmer et al. exploited this mechanism to engineer a closed loop therapeutic circuit by pairing HucR sensing with downstream uric acid degrading enzymes [53]. Optimized via integration of a KRAB repression domain, tandem *hucO* operator arrays, and the human uric acid transporter URAT1 for enhanced substrate uptake, the circuit achieved tunable, reversible responsiveness across human cell lines. Crucially, when implanted into uricase deficient mice, these engineered cells autonomously maintained circulating uric acid within physiological ranges and markedly attenuated renal urate crystal deposition (Fig. 2D). This HucR-based system represents a prototypical example of integrating metabolite sensing with therapeutic actuation, enabling autonomous regulation of disease-associated metabolites. Compared to conventional pharmacological approaches, such closed-loop systems offer the potential for sustained, precise, and self-adjusting treatment. However, challenges remain in ensuring long-term stability, biosafety, and precise control in complex *in vivo* environments, which are critical for clinical translation.

#### 2.4.4 Trimethylamine N-oxide (TMAO)

Trimethylamine N-oxide (TMAO) is a gut microbiota derived metabolite generated from dietary choline and carnitine [169]. Increasing evidence links elevated circulating TMAO levels to a broad spectrum of chronic diseases, including cardiovascular disease [[170], [171], [172]], chronic kidney disease [[173], [174], [175]], type 2 diabetes [[176], [177], [178]], and certain cancers [[179], [180], [181]]. In particular, TMAO promotes atherosclerosis and is strongly associated with adverse cardiovascular outcomes such as myocardial infarction and stroke [182]. Clinically, plasma TMAO levels correlate inversely with renal function and can independently predict mortality risk in patients with kidney disease [183], while also serving as a prognostic indicator in diabetic populations [184]. Despite its profound biomarker utility, current quantification relies strictly on endpoint measurements, hindering real time metabolic tracking and the development of personalized microbiome interventions.

In *Escherichia coli*, the atypical TorRST two component system controls TMAO respiration, where the periplasmic binding protein TorT and sensor kinase TorS detect extracellular TMAO to drive the phosphorylation of the response regulator TorR [185,186]. Zhang et al. engineered a highly sensitive TMAO biosensor based on TorRST by incorporating signal amplification cascades and optimized operator regions [55]. The platform exhibited strong correlation with commercial diagnostic kits. Capitalizing on this module, the authors constructed an intelligent probiotic strain, SEdegrT, integrating a sense transport degrade circuit. Upon sensing ambient TMAO, SEdegrT autonomously activates uptake and degradation pathways, reducing metabolic concentrations to below 50 μM within 24 h. This framework highlights the utility of converting pathogen derived sensory modules into probiotic therapeutic platforms, though establishing long term *in vivo* biocontainment and stability within complex gut environments remains paramount.

#### 2.4.5 Tetrathionate

Tetrathionate is a sulfur containing inorganic intermediate that accumulates exclusively in the inflamed intestine, generated when host derived reactive oxygen species oxidize endogenous thiosulfate [187]. Beyond serving as an inflammation marker, tetrathionate acts as a terminal electron acceptor that fuels the anaerobic expansion of pathogens like *Salmonella*, exacerbating microbial dysbiosis [23]. This inflammation specific production positions tetrathionate as a selective biomarker for monitoring inflammatory bowel disease (IBD) severity [188]. However, conventional detection methods are limited in their ability to monitor tetrathionate dynamically and in situ within the gut environment, restricting both mechanistic studies and real-time disease monitoring.

The membrane bound TtrSR two-component system from *Shewanella baltica* translates extracellular tetrathionate detection into transcriptional outputs via TtrS autophosphorylation and subsequent TtrR activation [189]. Daeffler et al. harnessed this architecture to construct a tetrathionate responsive biosensor in EcN, achieving non-invasive, real-time tracking of intestinal inflammation in murine colitis models [56]. This study demonstrated the feasibility of using engineered commensal bacteria as minimally invasive diagnostics for intestinal diseases. Expanding this sense and target capability, Wang et al. engineered a multifunctional strain coupling TtrSR sensing with motility control and the *ttrBCA* reductase cluster [57]. These engineered bacteria executed directed chemotaxis along tetrathionate gradients, utilizing the metabolite for localized colonization and growth advantages at inflamed sites. These advanced architectures demonstrate the potential of active disease localization, though clinical deployment requires strict verification of bacterial biocontainment, long term genetic stability, and the mitigation of off target colonization.

#### 2.4.6 N-acyl homoserine lactone (AHL)

N-acyl homoserine lactones (AHLs) are canonical quorum sensing signaling molecules employed by Gram negative bacteria to orchestrate population density dependent behaviors [190]. QS plays a critical role in the pathogenesis of numerous diseases by regulating virulence factor expression and biofilm formation, which underlies over 80% of chronic bacterial infections [191]. In particular, pathogens such as *Pseudomonas aeruginosa* and *Burkholderia cepacia* produce AHLs that are closely associated with persistent pulmonary infections in cystic fibrosis (CF) patients [192,193]. AHLs have therefore emerged as functional biomarkers of bacterial activity, reflecting not only the presence of pathogens but also their collective behavior and infection state [194,195]. In gastrointestinal disorders such as inflammatory bowel disease (IBD), microbial involvement and QS signaling have also been implicated in disease progression [196,197]. However, standard diagnostics rely on invasive biopsies or endoscopies and fail to capture real time microbial communication dynamics.

The LuxR family transcription factor TraR, encoded on the Ti plasmid of *Agrobacterium tumefaciens*, homodimerizes and binds target promoters strictly upon AHL ligation [198]. Chambers et al. developed an AHL biosensor leveraging the TraR framework to analyze mucopurulent respiratory secretions from CF patients [58]. The system achieved high sensitivity, quantifying diverse AHL species at ultra-low concentrations from minute sample volumes. This approach confirmed active *in vivo* quorum sensing and interspecies communication within infected lung microenvironments, bypassing the constraints of conventional low abundance analyte tracking. While AHL responsive biosensors offer an unprecedented functional lens into the active state of chronic infections, refining species specificity within polymicrobial niches and establishing robust, non-invasive delivery modalities represent the essential next steps for clinical translation.

Despite the broad development of genetically encoded biosensors, common performance bottlenecks persistently hinder their seamless clinical translation. These systems frequently face challenges including narrow dynamic ranges, high basal leakage, cross-reactivity with host metabolites, microenvironmental signal interference, and *in vivo* genetic instability. Overcoming these inherent limitations is essential for creating reliable clinical theranostics, making systematic optimization strictly necessary. The following section explores engineering strategies currently employed to resolve these bottlenecks and precisely tailor biosensor performance for complex physiological applications.

## 3 Optimization of genetically encoded biosensors

### 3.1 From signal transduction to output signals

Genetically encoded biosensors function as integrated signal-processing systems that convert molecular inputs into measurable outputs through a hierarchical workflow encompassing input recognition, signal sensing, information processing, and output generation. In the context of the applications discussed in Section 2, ranging from metabolic biomarker detection to theranostic circuits, the performance of these systems is ultimately determined not only by the sensing module itself but also by how efficiently signals are processed and translated into functional outputs.

Within this framework, the output module represents the final stage of signal transduction, where biochemical information is encoded into quantifiable or functional readouts. Transcription-based biosensors, when coupled with diverse reporter systems, can generate a wide spectrum of outputs tailored to specific applications. These outputs can be broadly categorized into three classes. First, optical outputs, including fluorescence and bioluminescence, enable real-time, quantitative, and often non-invasive monitoring; fluorescence-based systems are particularly suited for high-throughput screening [[199], [200], [201]], while bioluminescent systems offer high sensitivity without the need for external excitation [[202], [203], [204]]. Second, growth-based outputs convert molecular signals into selective cellular survival, for example through antibiotic resistance markers, enabling rapid enrichment or screening of target phenotypes [[205], [206], [207]]. Third, non-optical functional outputs, such as colorimetric enzyme reactions [208], electrical signals generated via microbial fuel cells [209,210], or acoustic signals mediated by gas vesicles [211], extend biosensor applications to low-resource settings, remote monitoring, and deep-tissue imaging. Importantly, these output modules are not merely passive reporters but can be integrated with synthetic gene circuits to enable signal amplification, logic computation, and actuation, thereby bridging sensing with downstream functions such as metabolic regulation or therapeutic response [18,19].

### 3.2 From quantitative description to system-level design

Genetically encoded biosensors function as core interfaces that translate molecular inputs into programmable cellular outputs, thereby enabling the integration of environmental sensing with synthetic gene circuits and downstream therapeutic responses. From a systems perspective, their performance is not solely determined by individual components but emerges from the coordinated operation of the entire input–sensing–processing–output cascade. Accordingly, optimizing biosensor function requires a unified framework that links quantitative system behavior to multi-layer circuit design.

The functional behavior of genetically encoded biosensors is quantitatively described by their dose–response relationship, which captures how variations in input signal concentration are transformed into output signal intensity. This relationship provides a generalizable basis for both evaluating and engineering biosensor performance. Several key parameters are widely used to characterize this response. Basal leakage represents the background output in the absence of input, reflecting intrinsic system noise. Dynamic range describes the maximal change in output relative to this baseline (i.e., fold induction), indicating signal amplitude. The response threshold (θ), often defined as the half-maximal effective concentration (EC50), determines the operational concentration window of the sensor. Response sensitivity, = ’ corresponding to the slope of the dose–response curve near the threshold, reflects how sharply the system responds to changes in input signal [212,213]. Together, these parameters define a multidimensional performance landscape in which biosensor behavior can be quantitatively interpreted.

While these parameters provide a descriptive framework, they also offer a direct entry point for rational engineering. Rather than being treated as independent metrics, basal leakage, dynamic range, threshold, and sensitivity can be mapped onto distinct layers of the input–sensing–processing–output cascade, enabling biosensor optimization to be reformulated as a process of systematically reshaping the dose–response landscape through coordinated control of sensing, signal transduction, and output generation. Within this systems-level view, biosensor engineering transitions from empirical tuning toward predictive and modular design.

#### 3.2.1 Dynamic range amplifying strategies

Dynamic range defines the maximal separation between OFF and ON states and determines the amplitude of signal output. This is largely governed by the gain of the output layer. Enhancing promoter strength or ribosome binding site (RBS) efficiency can significantly increase transcriptional and translational output levels [212,214], while increasing gene dosage through plasmid copy number or chromosomal integration further amplifies signal intensity [215]. More advanced designs employ multi-stage amplification architectures, such as transcriptional cascades, in which intermediate regulators amplify the initial sensing signal before activating the reporter gene, thereby producing nonlinear signal amplification and substantially widening the dynamic range. For example, employing the QF amplification circuit from the *Neurospora crassa* Q-system achieved a 3.5-fold increase in GFP fluorescence intensity. [[216], [217], [218]]. Wan et al. [219] constructed a cascade of mutually non-interfering orthogonal transcriptional amplifiers (HrpRS, RinAp80α, and ECF11987), which synergistically elevated output expression and resulted in a 200-fold enhancement of the output amplitude (Fig. 3A).

**Fig. 3:**
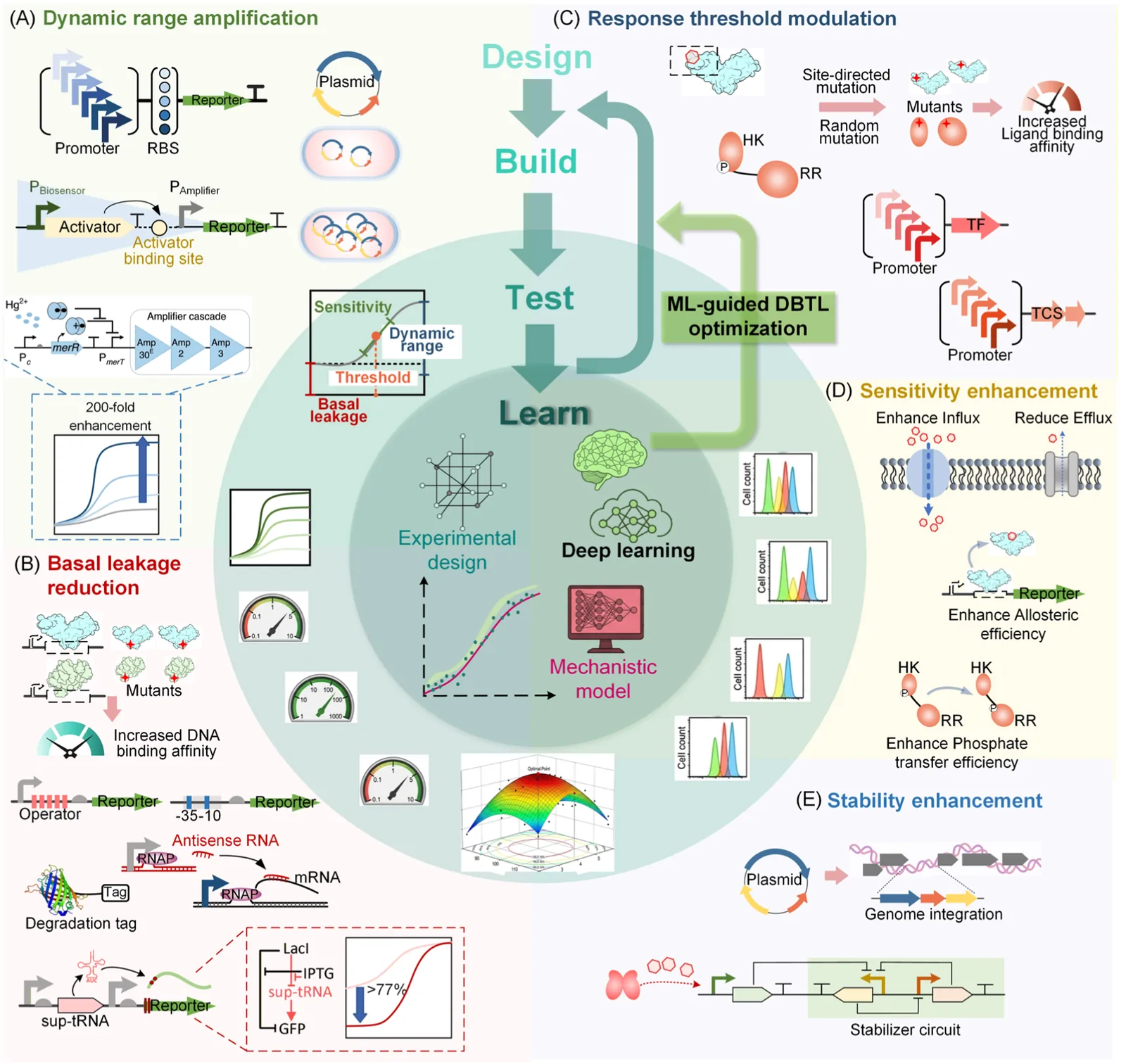
Comprehensive engineering strategies for optimizing the dose response landscape of genetically encoded biosensors as well as computational and machine learning approaches for sensor engineering, regulatory-element optimization, circuit design, and DBTL-guided iteration. The central diagram illustrates the interconnected performance parameters including sensitivity, dynamic range, response threshold, basal leakage, and stability. (A) Dynamic range amplification techniques involve increasing gene dosage, changing promoter and ribosome binding sites, or deploying multistage transcriptional cascades to maximize signal output. (B) Basal leakage reduction strategies minimize background noise by strengthening DNA binding affinities through directed mutations, optimizing core promoter sequences, or implementing posttranscriptional suppression via antisense RNA and degradation tags. (C) Response threshold modulation adjusts the operational sensing window by altering the ligand binding affinity of the regulatory module or by modifying the intracellular expression levels of the relevant transcription factors and two component systems. (D) Sensitivity enhancement focuses on steepening the response curve by increasing intracellular ligand concentrations through membrane transporter engineering, or by improving allosteric and phosphortransfer efficiencies. (E) System stability reinforcement ensures reliable long-term performance in complex environments through the chromosomal integration of genetic elements and the implementation of robust stabilizer circuits to buffer against external perturbations.

#### 3.2.2 Basal leakage reducing strategies

In parallel, minimizing basal leakage is essential for improving signal fidelity, as background expression reduces the distinguishability between inactive and activated states. Effective leakage control requires tightening regulatory constraints across multiple layers. A core strategy is to enhance the stringency of the regulatory module. This can be accomplished through multiple strategies. For example, site-directed mutagenesis can be employed to increase the binding affinity of a transcription factor (or a response regulator in a two-component system) to its cognate DNA operator sequence [220]. Alternatively, increasing the number of operator sites can more robustly repress downstream promoter activity in the ligand-free state. Concurrently, engineering the sequence of the inducible promoter itself, such as optimizing its −10 and −35 regions, can further reduce basal transcriptional activity without significantly compromising the maximum induced level [221]. Additional suppression can be implemented at the post-transcriptional and post-translational levels, for example through antisense RNA-mediated, suppressor transfer RNA-mediated, or degradation tags-mediated repression to accelerate reporter protein turnover [222]. For example, Wang et al. engineered suppressor tRNAs charged with canonical amino acids in a LacI-based biosensor, this suppressor tRNA regulation reduced background leakage by more than 77% [223]. These methods often require combination and fine-tuning according to the specific sensor architecture to maintain high inducibility and dynamic range while minimizing basal leakage (Fig. 3B).

#### 3.2.3 Response threshold modulating strategies

Another critical dimension of biosensor optimization lies in tuning the response threshold, which determines the concentration range over which the biosensor operates. Because native sensing ranges often do not align with physiologically relevant concentrations, threshold adjustment is frequently required for practical applications. The most direct approach is to alter the ligand-binding affinity of the sensing module. For biosensors based on allosteric transcription factors (aTFs), site-saturation mutagenesis or directed evolution of the ligand-binding domain can be employed to screen for variants with stronger or weaker binding capabilities, thereby lowering or raising the ligand concentration required for activation [224]. Another effective strategy is to modulate the intracellular concentration of the sensing element itself. For instance, adjusting the expression level of the transcription factor by altering the strength of its own promoter can systematically shift the dose-response curve [[225], [226], [227]]. In two-component systems, adjusting the balance between kinase and phosphatase activities of the sensor histidine kinase, or modulating the expression level of the response regulator, provides additional means to shift the activation threshold [228,229] (Fig. 3C).

#### 3.2.4 Detection sensitivity enhancing strategies

Enhancing sensitivity aims to enable the sensor to produce a steeper and more pronounced output response to minute changes in ligand concentration. A key strategy is to optimize the overall transduction efficiency from signal perception to signal output. A fundamental yet effective method is to increase the effective intracellular concentration of the ligand. This can be achieved by heterologously expressing or overexpressing specific membrane transporters for the molecule to enhance its influx, or by knocking out efflux transporters to prevent its loss [230,231]. Furthermore, engineering the sensing protein (e.g., a transcription factor) to optimize the efficiency of its conformational change upon ligand binding, or optimizing the phosphotransfer kinetics between the kinase and the regulator in a TCS, holds promise for enabling the system to convert input signals into output signals with higher “gain,” thereby yielding a sharper response curve. Zhu et al. [232] addressed the insufficient sensitivity of conventional mercury biosensors by evolving transcription factor MerR. The resulting mutant m4-1achieved a limit of detection of 0.313 ng/L—a 115-fold improvement over the wild-type. (Fig. 3D).

#### 3.2.5 System stability reinforcing strategies

Ensuring system stability is critical for translating biosensors from controlled laboratory environments to real-world applications. Chromosomal integration of the sensing circuit can eliminate variability associated with plasmid maintenance and copy number fluctuations, thereby improving long-term reliability. At the circuit level, incorporating robust network topologies, such as incoherent feedforward loops, can buffer environmental perturbations and maintain consistent output across varying conditions [233]. For applications requiring live cells, cell encapsulation and preservation techniques become necessary. Examples include encapsulating engineered bacteria within hydrogel-elastomer hybrid materials or processing them into stable formulations via lyophilization. These methods effectively protect cell viability, enabling reliable detection functionality even in complex environments or after long-term storage [25] (Fig. 3E).

Taken together, these design dimensions define an interconnected optimization landscape in which biosensor performance emerges from coordinated tuning across multiple layers of the input–sensing–processing–output cascade. Importantly, this framework establishes a direct conceptual bridge between molecular sensing and higher-order circuit function. By integrating optimized biosensors with synthetic gene circuits, it becomes possible to construct systems that not only detect specific biomarkers but also process this information and trigger context-dependent responses. Such “sense–process–respond” architectures underpin the development of closed-loop therapeutic systems, in which real-time molecular inputs are continuously translated into adaptive interventions. In this way, advances in biosensor optimization extend beyond improved detection, ultimately enabling the rational design of intelligent cellular systems for diagnostics, metabolic control, and precision medicine.

### 3.3 Computational and machine learning-guided biosensor optimization

Over the past few decades, the engineering of genetically encoded biosensors has undergone three paradigm shifts: from early empirical trial-and-error assembly, to semi-rational design relying on structural modeling and high-throughput screening, and ultimately to the current era of predictive design driven by artificial intelligence (AI), particularly machine learning (ML) [234,235]. Currently, ML are profoundly reshaping the “Design-Build-Test-Learn” (DBTL) cycle in synthetic biology, propelling sensor engineering from the traditional trial-and-error paradigm toward data driven precision customization [236]. This transformation facilitates biosensor development, the optimization of regulatory elements, complex genetic circuit design, and the accelerated DBTL-guided iteration.

Traditional biosensor development relies heavily on the empirical combination of limited natural components, and complex context dependency often renders system performance unpredictable. ML has shifted this paradigm by mining high dimensional sequence-function data, enabling the rational prediction and design of sensing modules. For instance, Zhou et al. [237] developed a high-throughput workflow integrating trackable DNA assembly and FACS-seq for the massively parallel construction and characterization of thousands of biosensors. After constructing a FapR-fapO-based malonyl-CoA biosensor library containing 5184 combinations and characterizing 2632 of them, the study applied the XGBoost algorithm to successfully predict the genotype-phenotype relationships of the uncharacterized combinations, generating a global landscape of the combinatorial space. This strategy not only yielded high-performance sensors with a dynamic response range up to 6.38 but also revealed the critical impact of recognition sequence insertion schemes and upstream enhancers on the dynamic range. It proved that data-driven modeling can effectively replace tedious physical screening to achieve the efficient customization of sensor performance.

Once the core sensing module is determined, the performance of the regulatory elements directly dictates the final signal output range of the sensor. Faced with a vast genotypic space, ML can bypass time-consuming exhaustive screening to achieve precise optimization and even *de novo* design of regulatory elements. Ding et al. [238] developed an efficient, deep learning-based strategy for element optimization. The team utilized high-throughput flow cytometry and next-generation sequencing to acquire “sequence-phenotype” data for 12,000 cross-ribosome binding sites (cRBSs), and thereby trained a convolutional neural network (CNN)-based classification model (CLM-RDR). This model achieved a prediction accuracy of 0.86 for the dynamic range of sensors under different RBS combinations and demonstrated excellent generalizability across multiple metabolite biosensors. Subsequently, the team further established a forward-reverse engineering platform. In forward engineering, the WGAN-GP model was employed for the *de novo* design of cRBS sequences, achieving a dynamic range prediction accuracy of up to 98% when combined with a CNN model; in reverse engineering, by utilizing a generative platform integrating a sequence autoencoder with BAGAN-GP, they successfully enabled the reverse customization of ideal cRBS sequences based on target dynamic ranges. This bidirectional design closes the loop from “genotype to phenotype” and “phenotype to genotype,” providing a novel paradigm for the customized development of regulatory elements [239].

At the systems engineering level of genetic circuit design, ML can not only guide the design of individual elements but also translate massive amounts of experimental data into actionable global design principles, thereby guiding the design of multi-level genetic circuits and significantly streamlining the DBTL cycle. Graham et al. [240] developed a high-throughput microfluidic platform named “Dynomics,” enabling the cultivation and monitoring of 2176 *E. coli* micro-communities and the high-resolution tracking of genome scale transcriptional dynamics. By screening strains containing 1807 unique promoter-GFP reporters and assaying multiple heavy metal stresses, researchers generated high-resolution temporal gene expression data. Researchers effectively overcame the “black-box” limitations of traditional single-point response designs and decoded multi-dimensional synergistic response features from dynamic transcriptional networks. Furthermore, to optimize complex systems with minimal data, Pandi et al. [241] developed the METIS active machine learning workflow centered on the XGBoost algorithm, which aims to optimize genetic and metabolic networks using extremely limited experimental data. In the optimization of a LacI-based multi-level gene regulatory circuit, this platform not only improved the activity by two orders of magnitude but also precisely pinpointed and overcame the bottleneck of plasmid resource competition through feature importance analysis. In a more complex application of in vitro metabolic networks, targeting a synthetic CO_2_ fixation pathway, the platform successfully achieved optimization within a theoretical combinatorial space of 10^25^ conditions using only 1000 experiments, thereby increasing productivity tenfold. The METIS workflow not only achieves targeted optimization but also quantitatively assesses the relative importance of individual components to the overall network, opening up a novel data-driven avenue for identifying unknown interactions and rate-limiting steps in complex biological networks.

## 4 From genetically encoded biosensors to clinical medical devices

Genetically encoded biosensors are driving a paradigm shift from static, ex vivo measurements toward integrated, real-time, and ultimately closed-loop biomedical systems. By coupling molecular sensing with programmable gene circuits and diverse output modalities, these technologies are redefining how biological information is detected, interpreted, and acted upon. This transition can be broadly organized into three progressively integrated translational modalities: point-of-care diagnostics, wearable monitoring systems, and engineered living therapeutics, representing a continuum from discrete measurement to continuous sensing and, ultimately, autonomous intervention.

### 4.1 Point-of-care diagnostic devices

The integration of genetically encoded biosensors into point-of-care (POC) platforms is transforming rapid diagnostics from single-analyte detection into programmable molecular analysis systems. Conventional POC devices, exemplified by personal glucose meters, rely on predefined biochemical reactions to quantify specific analytes. In contrast, biosensor-enabled systems introduce a layer of biological computation, allowing detection of complex molecular signatures through engineered gene circuits. This paradigm shift democratizes precision medicine by migrating sophisticated analytical capabilities from centralized laboratories to remote clinics and domestic environments. Employing lyophilized acellular formats removes traditional dependencies on living cultures and refrigerated supply networks. These exceptionally stable sensors require only hydration for activation, maximizing diagnostic feasibility in environments with limited resources. Consequently, this innovation establishes a novel framework for intelligent and adaptive diagnostic systems deployable on demand. The current research landscape fuses biological intelligence with practical engineering hardware to transition laboratory prototypes into clinical tools, with the primary objective of leveraging synthetic biology to detect complex biomarkers through inexpensive portable devices for immediate local analysis.

A key strategy in this field involves interfacing biosensors with established electronic readout platforms. Electrochemical devices, particularly glucose meters, provide a robust and widely accessible infrastructure for signal quantification [242]. Building on this paradigm, Amalfitano et al. [243] developed a biosensor based on the TetR/TetO system compatible with personal glucose meters, enabling detection of diverse targets including tetracycline, *Salmonella Typhi* RNA, and SARS-CoV-2 RNA. The sensor performance was successfully validated in simulated serum samples, and its results for clinical samples showed high concordance with RT-qPCR. This research established a connection between synthetic biology diagnostic tools and glucose meters, offering a solution with significant potential for rapid, low-cost disease detection in resource-limited areas.

In parallel, paper-based microfluidic platforms offer an alternative route toward decentralized diagnostics. By integrating lyophilized, cell-free biosensor systems into patterned porous substrates, these devices enable capillary-driven sample processing and visual signal output. Free et al. [244] demonstrated a glutamine biosensor embedded in a freeze-dried in vitro system that can be stored at ambient temperature and activated upon hydration, producing a colorimetric readout within 30 min. Such designs eliminate cold-chain dependence and reduce operational complexity, making them particularly suitable for resource-limited settings. This work provides a scalable technical paradigm for developing POC tests targeting various disease biomarkers. By placing a suitable genetically encoded biosensor into a working system, lyophilizing the system, and combining it with an appropriate paper-based platform, genetically encoded biosensors can be transformed into truly portable, user-operated diagnostic tools.

Collectively, these approaches illustrate how genetically encoded biosensors can be coupled with simple, low-cost hardware to enable rapid, on-site molecular diagnostics. Current developments have already demonstrated portable detection of antibiotic residues [245], viral RNA [246], mRNA [247], heavy metal ions [248], and disease biomarkers [249], including multiplexed sensing capabilities. However, challenges remain in improving robustness against complex sample matrices and further simplifying user workflows to enable widespread adoption.

### 4.2 Wearable devices for continuous monitoring

While POC devices provide discrete measurements, wearable biosensor systems extend this capability to continuous and longitudinal monitoring of physiological states [250]. The evolution of such wearable systems marks a critical step toward applying sophisticated gene circuits to personalized preventive medicine [251,252]. Advances in flexible materials, microfluidics, and miniaturized electronics have enabled non-invasive sampling of biofluids such as sweat, saliva, tears, and interstitial fluid, supporting real-time analysis of biomarkers including glucose, lactate, and cortisol [253,254]. Concurrently, advancing device integration with multiparameter sensing and wireless transmission supports personalized healthcare, while embedding genetically encoded biosensors into wearable platforms further elevates device capabilities through programmable molecular sensing.

Nguyen et al. [255] demonstrated the incorporation of gene circuit-based biosensors into flexible substrates, including elastomers and functional textiles, forming wearable analytical patches. These systems utilize microfluidic structures for controlled sample handling and employ lyophilized biosensing components that are activated upon hydration. Detection outputs, including colorimetric, fluorescent, and luminescent signals, can be transmitted through integrated optical systems, enabling real-time readout. This design paradigm exemplifies how biological sensing modules can be standardized into “lyophilize-and-activate” formats, facilitating their integration into portable and user-friendly devices. Importantly, wearable biosensors shift diagnostics from episodic testing to continuous monitoring, enabling dynamic tracking of disease progression and treatment response.

From a translational perspective, the rapid expansion of the wearable biosensor market underscores its clinical and commercial potential [256]. The primary drivers in this field stem from rising healthcare demands and the intelligent integration with consumer electronics. The convergence of biosensing, materials science, and consumer electronics has led to widespread adoption of devices such as smartwatches, patches, and contact lenses for health monitoring [257]. Currently, numerous technology and healthcare companies have launched related products covering various monitoring dimensions including blood glucose, electrocardiography, and blood oxygen saturation. Although current systems face challenges related to signal accuracy, user compliance, and data security, the integration of genetically encoded biosensors with artificial intelligence and data analytics is expected to further enhance their role in personalized and preventive medicine.

### 4.3 Engineered probiotics for monitoring and therapy

The most advanced stage of biosensor translation is represented by engineered living therapeutics, in which genetically encoded biosensors are embedded within probiotic microorganisms to enable in situ sensing and response. This approach transforms passive microbial therapies into active, programmable systems capable of operating directly within the host environment. First defined in 1965 as microbial secretions promoting the growth of other organisms [258], probiotics have become fundamental to clinical health interventions. They are broadly classified into traditional and nontraditional categories. Traditional strains, including lactic acid bacteria, *Escherichia coli Nissle 1917*, and *Saccharomyces boulardii*, possess established safety profiles. Conversely, nontraditional species such as *Akkermansia muciniphila*, *Faecalibacterium prausnitzii*, *Bacteroides fragilis*, and *Eubacterium hallii* demonstrate novel potential in maintaining gut homeostasis and synthesizing short-chain fatty acids [259]. Clinically, probiotics mitigate diseases including inflammatory bowel disease, cancer, obesity, and diabetes through microbiome modulation, immune enhancement, pathogen inhibition, and metabolic regulation [[260], [261], [262]]. However, traditional probiotics often face limitations such as weak colonization capacity and variable efficacy in practical applications, driving the development of next-generation probiotic technologies.

A central concept in this field is the construction of theranostic circuits, in which detection of disease-associated biomarkers triggers targeted therapeutic outputs [263,264]. Lubkowicz et al. [265] engineered a probiotic *Lactobacillus reuteri* strain incorporating a biosensor based on the agr quorum-sensing system to detect *Staphylococcus aureus* autoinducing peptide (AIP-I), enabling sensitive and specific detection of infection signals. This engineered probiotic offers a simpler and more cost-effective alternative to traditional methods like mass spectrometry. Similarly, Mao et al. [266] engineered *Lactococcus lactis* to function as a living diagnostic tool for cholera. By integrating *Vibrio cholerae* CqsS domains into the native NisK-NisR two component system, the probiotic successfully detects the specific quorum-sensing signal molecule of *V. cholerae* for noninvasive fecal diagnostics. Because *L. lactis* naturally secretes lactic acid to inhibit *V. cholerae*, its administration in a neonatal mouse model significantly reduced intestinal bacterial loads and enhanced survival.

Beyond diagnostics, biosensor-enabled probiotics can execute targeted therapeutic functions. Tetrathionate, a biomarker of intestinal inflammation, has been used to construct engineered *E. coli* Nissle 1917 strains capable of sensing inflammatory states [57]. Building on this, Palmer et al. [24] developed a novel engineered probiotic by integrating the gene for the synthetic antimicrobial peptide Microcin H47. This strain detects the intestinal inflammation signal tetrathionate to initiate a specific antibacterial program against *Salmonella*. Subsequent in vitro testing demonstrated that within a simulated inflamed intestinal environment, the engineered probiotic maintained a significant fitness advantage while effectively inhibiting *Salmonella* growth. Similarly, Koh et al. [267] engineered *Escherichia coli* Nissle 1917 with a dual function gene circuit to sense and restrict *Clostridioides difficile* infections prophylactically. Because the pathogenesis depends heavily on bile acid dysregulation caused by gut microbiome imbalance, the system utilizes a biosensor to detect dysbiosis triggered by antibiotics. Upon activation, an integrated execution module expresses bile acid metabolism genes to locally convert excess taurocholic acid into cholic acid. This targeted metabolic intervention inhibits *C. difficile* spore germination, suppresses bacterial proliferation, and decreases toxin concentrations. Following successful in vitro validation, prophylactic administration in a murine model significantly alleviated clinical symptoms, prevented intestinal tissue damage, and reduced pathogen colonization. These systems exemplify the transition from open-loop to closed-loop therapeutic paradigms.

Numerous biotechnology initiatives currently explore engineered probiotics for immune and metabolic therapy. Synlogic demonstrated this potential through *Escherichia coli* Nissle 1917 strains like SYN1618 and SYN1934 for phenylketonuria [68], alongside various candidates for other metabolic conditions and cancer [[268], [269], [270], [271]]. While these clinical endeavors underscore broad therapeutic promise, they also highlight significant translational challenges. Furthermore, these initial platforms operated as static open loop systems lacking inducible biosensors, leading to constitutive metabolite degradation without regulatory control. Transitioning to an intelligent closed loop paradigm requires integrating genetically encoded biosensors into a colonizing probiotic chassis. This advancement allows the therapeutic system to autonomously monitor real time pathophysiological signals, thereby facilitating precise, personalized, and dose adaptive dynamic treatment.

Engineered probiotics, as living therapeutics, enable real-time sensing of and dynamic responses to biomarkers through the construction of theranostic circuits. By linking biomarker detection to therapeutic response, these systems establish autonomous interventions that can adapt to fluctuating physiological conditions in real time. Despite their considerable therapeutic potential, clinical translation still faces multiple challenges. First, *in vivo* colonization efficiency of engineered probiotics remains low, making it difficult to compete within the complex gut microenvironment. Second, biosafety is a paramount concern, including the risks of horizontal gene transfer and unintended environmental persistence. To mitigate these risks, stringent biocontainment mechanisms like auxotrophic dependencies or programmed suicide switches must be integrated into the chassis. Finally, early-stage clinical trials have revealed issues such as microbiome perturbation and insufficient durability of the genetic circuits. Overcoming these hurdles will require closer synergy between synthetic circuit robustness, host chassis engineering, and rigorous clinical trial design.

Taken together, the progression from POC diagnostics to wearable monitoring systems and engineered living therapeutics illustrates a continuous trajectory in the clinical translation of genetically encoded biosensors. At one end of this spectrum, biosensors enhance the accessibility and depth of molecular diagnostics; at the other, they enable autonomous, closed-loop therapeutic systems capable of real-time decision-making and intervention within the body. This evolution reflects a broader shift from passive measurement to active biological control. By integrating sensing, computation, and actuation within programmable biological systems, genetically encoded biosensors provide a foundational platform for next-generation medical technologies. Future advances in circuit design, host engineering, and device integration will further accelerate this transition, ultimately enabling seamless convergence between diagnostics and therapy in precision medicine.

## 5 Conclusion and perspectives

Genetically encoded biosensors are redefining the interface between biological sensing and medical intervention, enabling a transition from static measurements to dynamic, programmable, and increasingly autonomous systems. By coupling real-time detection of disease-associated biomarkers with engineered gene circuits, these platforms establish a foundation for “sense–process–respond” architectures that integrate diagnosis and therapy within a unified framework. Such systems not only enhance the spatiotemporal precision of intervention but also point toward a broader shift from reactive treatment paradigms to predictive and preventive healthcare.

Looking forward, addressing the inherent limitations of transcriptional biosensors relative to post-transcriptional and post-translational sensors is a key frontier in synthetic biology. As highlighted in this article, while transcriptional sensors (aTFs and TCSs) excel at executing complex logic operations and driving high-level expression of therapeutic actuators, their response dynamics are fundamentally constrained by the multi-step processes of transcription and translation. In contrast, protein- or RNA-based sensors offer near-instantaneous signal transduction, but often at the expense of lower signal gain or limited actuation capacity. To bridge these performance gaps, the lineage of next generation intelligent biological systems will transcend traditional transcription factor paradigms by incorporating rapidly evolving molecular tools. Specifically, clustered regularly interspaced short palindromic repeats (CRISPR) based biosensors offer unprecedented nucleic acid recognition capabilities with single nucleotide specificity, while programmable RNA regulators, such as toehold switches and riboswitches, enable versatile post transcriptional control with minimal genetic footprints. To bypass the historical liabilities of cell toxicity, low transfection efficiency, and stringent biological containment, cell free synthetic biology has emerged as a high-fidelity deployment platform. Operating outside living boundaries, cell free platforms preserve complex enzymatic networks within paper based or polymeric matrices, providing robust, shelf stable point of care diagnostics that bridge the gap between abstract synthetic networks and pragmatic clinical deployment.

Concurrently, the continued development of this field will likely be driven by the convergence of synthetic biology with data-driven methodologies, particularly artificial intelligence. The large-scale, longitudinal datasets generated by biosensor-enabled monitoring systems create new opportunities for extracting clinically actionable insights. Deep learning approaches may facilitate the interpretation of complex, multidimensional biological signals, enabling predictive modeling of disease progression, therapeutic response, and patient-specific risk profiles. In this context, biosensors can be viewed not only as measurement tools but also as integral components of adaptive medical systems, in which sensing and decision-making are tightly coupled.

Beyond data analysis, computational approaches are expected to play an increasingly important role in biosensor design and optimization. Integration of multi-omics datasets may accelerate the identification of clinically relevant biomarkers and guide the engineering of sensing modules with improved specificity, sensitivity, and tunability. The historical reliance on trial-and-error screening is increasingly replaced by artificial intelligence assisted circuit design, where deep learning algorithms predict protein conformation, optimize promoter strength, and mitigate genetic cross talk before experimental execution. In parallel, advances in protein design, regulatory circuit engineering, and host chassis development will further expand the functional scope of biosensors, enabling detection of increasingly complex physiological states (Fig. 4).

**Fig. 4:**
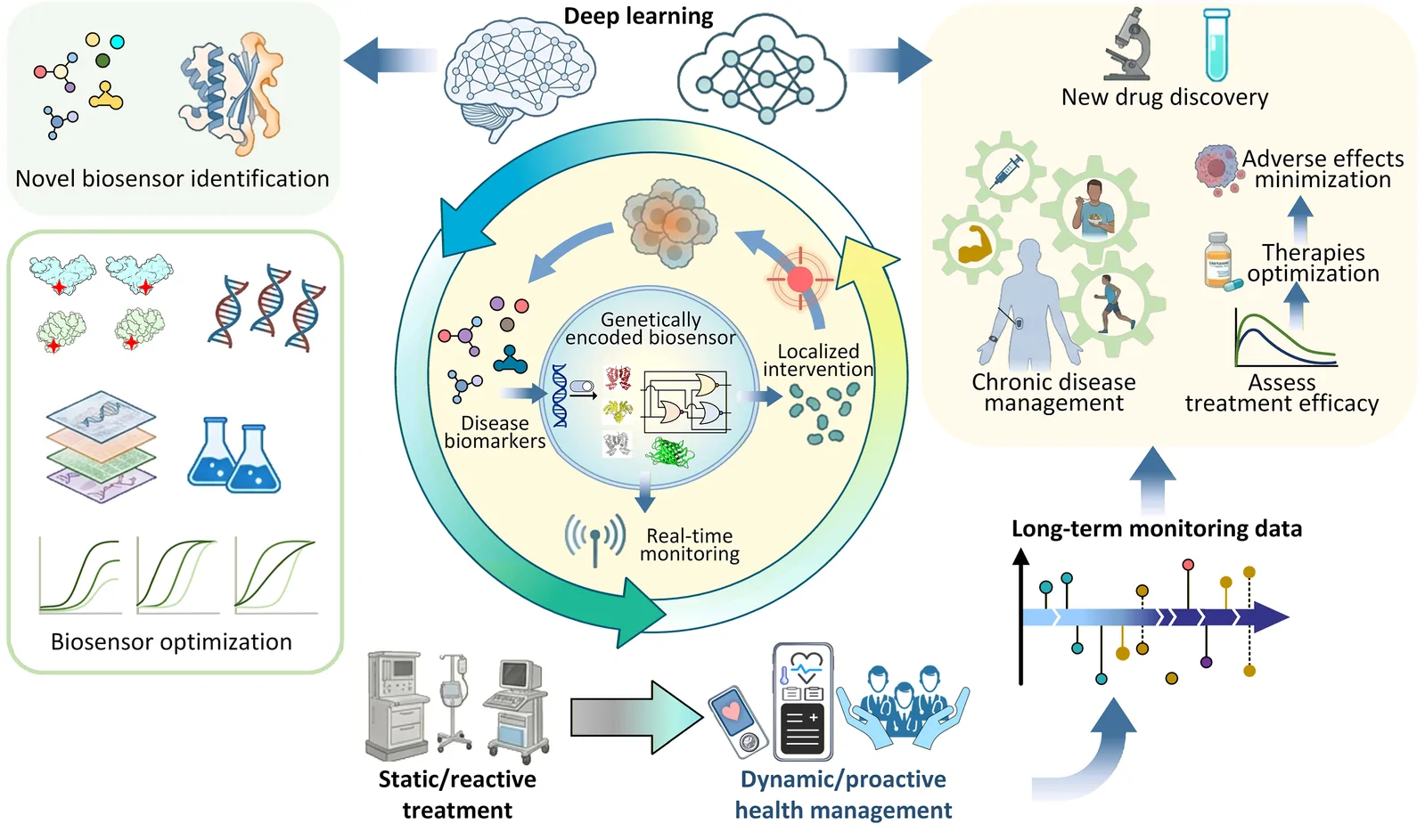
Future perspectives and paradigm shifts enabled by genetically encoded biosensors integrated with artificial intelligence. The central schematic illustrates the continuous sense process respond cycle where engineered biosensors detect disease biomarkers in real time to deploy precise localized interventions. On the left deep learning methodologies accelerate the identification of novel sensing modules and streamline systematic optimization processes including protein engineering and genetic circuit design. The bottom section highlights how this technological evolution drives a fundamental transition from traditional static reactive treatments toward dynamic proactive health management utilizing portable platforms. On the right accumulated long term monitoring data empowers comprehensive chronic disease management and accelerates new drug discovery. By continuously assessing treatment efficacy these data driven frameworks facilitate therapy optimization and adverse effect minimization ultimately realizing personalized and predictive medicine.

Despite these advances, significant challenges remain in translating genetically encoded biosensors into clinical practice, including issues related to robustness in complex biological environments, long-term safety, regulatory approval, and standardization of device platforms. Addressing these challenges will require coordinated efforts across disciplines, spanning synthetic biology, materials science, data science, and clinical research. Ultimately, genetically encoded biosensors represent more than a class of analytical tools; they constitute a foundational technology for constructing integrated, intelligent, and adaptive biomedical systems. As these platforms continue to evolve, they are poised to enable a new generation of precision medicine strategies in which sensing, computation, and intervention are seamlessly interconnected.

## CRediT authorship contribution statement

**Jianping Xu:** Writing – review & editing, Writing – original draft. **Qingsheng Qi:** Supervision. **Qian Wang:** Writing – review & editing, Supervision, Project administration, Funding acquisition.

## Data availability

No data was used for the research described in the article.

## Declaration of competing interest

The authors declare that they have no known competing financial interests or personal relationships that could have appeared to influence the work reported in this paper.
